# Transpulmonary thermodilution-derived cardiac function index identifies cardiac dysfunction in acute heart failure and septic patients: an observational study

**DOI:** 10.1186/cc7994

**Published:** 2009-08-11

**Authors:** Simon Ritter, Alain Rudiger, Marco Maggiorini

**Affiliations:** 1 Intensive Care Unit, Department of Internal Medicine, Triemli City Hospital, Birmensdorferstrasse 497, CH-8063 Zurich, Switzerland; 2 Intensive Care Unit, Department of Internal Medicine, University Hospital Zurich, Raemistrasse 100, CH-8091 Zurich, Switzerland

## Abstract

**Introduction:**

There is limited clinical experience with the single-indicator transpulmonary thermodilution (pulse contour cardiac output, or PiCCO) technique in critically ill medical patients, particularly in those with acute heart failure (AHF). Therefore, we compared the cardiac function of patients with AHF or sepsis using the pulmonary artery catheter (PAC) and the PiCCO technology.

**Methods:**

This retrospective observational study was conducted in the medical intensive care unit of a university hospital. Twelve patients with AHF and nine patients with severe sepsis or septic shock had four simultaneous hemodynamic measurements by PAC and PiCCO during a 24-hour observation period. Comparisons between groups were made with the use of the Mann-Whitney *U *test. Including all measurements, correlations between data pairs were established using linear regression analysis and are expressed as the square of Pearson's correlation coefficients (*r*^2^).

**Results:**

Compared to septic patients, AHF patients had a significantly lower cardiac index, cardiac function index (CFI), global ejection fraction, mixed venous oxygen saturation (SmvO_2_) and pulmonary vascular permeability index, but higher pulmonary artery occlusion pressure. All patients with a CFI less than 4.5 per minute had an SmvO_2 _not greater than 70%. In both groups, the CFI correlated with the left ventricular stroke work index (sepsis: *r*^2 ^= 0.30, *P *< 0.05; AHF: *r*^2 ^= 0.23, *P *< 0.05) and cardiac power (sepsis: *r*^2 ^= 0.39, *P *< 0.05; AHF: *r*^2 ^= 0.45, *P *< 0.05).

**Conclusions:**

In critically ill medical patients, assessment of cardiac function using transpulmonary thermodilution technique is an alternative to the PAC. A low CFI identifies cardiac dysfunction in both AHF and septic patients.

## Introduction

Several studies have suggested that there is no clear benefit, or that there may even be harm, in using a pulmonary artery catheter (PAC) in critically ill patients [1-4]. As a result, the use of PAC decreased substantially over the last decade [5]. However, PAC is still recommended for the hemodynamic monitoring of critically ill patients with heart failure [6] because it allows the assessment of the pulmonary artery occlusion pressure (PAOP), which may provide information on left ventricular function [7]. As an alternative to the more invasive PAC, the use of the transpulmonary thermodilution method (pulse contour cardiac output, or PiCCO) has been suggested [8]. The PiCCO monitor measures cardiac output (CO) and the global end-diastolic volume indexed for body surface area (GEDVI) as well as parameters of cardiac performance such as the cardiac function index (CFI) and the global ejection fraction (GEF). It also provides an estimate of extravascular lung water (EVLW) and calculates the pulmonary vascular permeability index (PVPI) [9-11], which allows the differentiation between a hydrostatic and a permeability type of pulmonary edema [12,13]. Volumetric parameters better estimate preload than central venous pressure or PAOP [8,10,11,14], and EVLW monitoring is of prognostic relevance [15-18]. A recent study suggested that guiding fluid and catecholamine therapy by an algorithm based on GEDVI and EVLW reduces postoperative vasopressor and catecholamine requirements in cardiac surgery patients [19].

The PiCCO method has been validated mainly in surgical patients and, to a lesser extent, in patients with sepsis [14,20-22]. However, there is still limited clinical experience with PiCCO-derived parameters of cardiac function and volume status in critically ill medical patients, particularly in those with acute heart failure (AHF) [8]. Therefore, we retrospectively analysed a series of simultaneous measurements by PiCCO and PAC in patients with AHF, severe sepsis, or septic shock.

## Materials and methods

### Study design

The study was performed at the 12-bed medical intensive care unit (ICU) of the University Hospital Zurich, Switzerland. Approval was given by our Institutional Review Board. Due to the retrospective nature of the analysis, the need for informed consent was waived. Twenty-one patients (15 males and 6 females) with circulatory failure monitored with a PAC were included in the study. Treatment was directed by the clinicians in charge of the patients. In 17 patients (81%), PAC was inserted within 1 day after ICU admission. After initial hemodynamic stabilisation but before removal of the PAC, the arterial line was switched to a PiCCO catheter in order to have less invasive continuous monitoring of CO for vasopressor weaning and fluid management. This provided a unique opportunity of simultaneous monitoring with the two methods during a 24-hour period. During this period, the dosage of vasoactive drugs was progressively decreased and volume was substituted or removed according to the clinical treatment strategy. Simultaneous recordings started 2 days (interquartile range [IQR] 1 to 4 days) after ICU admission. In each patient, four consecutive measurements were performed before PAC removal. Median (IQR) time intervals from baseline to the second and third measurements were 5 (4 to 8) and 13 (9 to 16) hours, respectively. All four measurements were realised after 19 (14 to 22) hours. A total of 84 simultaneous hemodynamic measurements were recorded and finally analysed.

### Patient characteristics

Severe sepsis was defined according to the published guidelines as systemic inflammatory response syndrome with infection associated with organ dysfunction [23]. AHF was diagnosed in the presence of an underlying heart disease and congestive heart failure, pulmonary edema, or cardiogenic shock [6]. Diagnosis of AHF was based on clinical signs of congestion (dyspnea, orthopnea, rales, or elevated jugular venous pressure), low CO with organ hypoperfusion, and bilateral alveolar consolidations on chest x-ray. Echocardiography and coronary angiography were performed only when clinically indicated. The severity of illness was described by the Simplified Acute Physiology Score (SAPS II) as calculated with the worst values within 24 hours following ICU admission [24].

AHF and severe sepsis or septic shock were diagnosed in 12 and 9 patients, respectively. Baseline characteristics on ICU admission are shown in Table 1. Coronary heart disease was present in 7 patients with AHF. Other underlying heart diseases included dilatative cardiomyopathy (n = 2), non-compaction cardiomyopathy (n = 1), valvular heart disease (n = 1), and congenital heart disease (n = 1). Cardiac imaging by echocardiography or coronary angiography or both was available in all heart failure patients except in two with known ischemic heart disease. The septic patients suffered from proven bacterial infection, namely pneumonia (n = 6), abdominal infection (n = 1), urogenital tract infection (n = 1), and puerperal sepsis (n = 1). Twelve patients (57%) required norepinephrine (0.1 to 0.3 μg/kg per minute), 13 patients (62%) needed inotropic support with dobutamine (1.5 to 6 μg/kg per minute), milrinone, or levosimendan, and 6 patients (29%) received intravenous vasodilatators such as nitroglycerin or nesiritide. Furthermore, 16 patients (76%) were mechanically ventilated, and 11 patients (52%) had renal replacement therapy by continuous veno-venous hemofiltration.

**Table 1:** Baseline characteristics of patients on admission to the intensive care unit

	Sepsis n = 9	Acute heart failure n = 12	*P *value
Gender, male/female	5/4	10/2	0.33
Age, years	52 (37–65)	65 (55–71)	0.15
Body mass index, kg/m^2^	23.4 (20.4–25.5)	25.0 (23.3–29.7)	0.19
SAPS II	49 (41–63)	35 (27–65)	0.19
PaO_2_/FiO_2 _ratio, mm Hg	165 (115–206)	254 (210–377)	0.004
ProBNP, ng/L	13,104 (6,964–27,225)	13,822 (7,692–23,885)	0.92
Troponin T, μg/L	0.03 (0.01–0.40)	0.11 (0.03–2.57)	0.42
Creatinine, μmol/L	129 (97–215)	138 (114–191)	0.65
White blood cell count, per mm^3^	12.5 (9.7–18.8)	9.5 (7.3–12.6)	0.19
CRP, mg/L	244 (85–334)	28 (7–150)	0.006
PCT, ng/mL	18.0 (2.5–34.4)	0.3 (0.2–1.3)	0.001
LVEF^a^, percentage	60 (50–65)	28 (19–43)	0.005

Data are presented as numbers or medians (interquartile ranges). Creatinine, norm 70 to 105 μmol/L. Troponin T, norm <0.10 μg/L. ^a^Echocardiography was performed in seven patients with sepsis and in nine patients with acute heart failure. CRP: C-reactive protein (norm <5 mg/L); FiO_2_: inspired oxygen fraction; LVEF: left ventricular ejection fraction; PaO_2_: partial arterial oxygen pressure; PCT: procalcitonin (norm <0.5 ng/mL); ProBNP: N-terminal pro-B-type natriuretic peptide (norm <227 ng/L); SAPS II: Simplified Acute Physiology Score II.

### Hemodynamic measurements

A continuous CO thermodilution PAC (model VIP 139F75; Edwards Lifesciences LLC, Irvine, CA, USA) was inserted via a central vein into the right pulmonary artery. Correct placement of the catheter was checked by appropriate pressure traces and fluoroscopy. The PAC was used for measurements of pulmonary artery pressure, PAOP, and cardiac index. Special care was taken to define the zero reference at midchest level and to perform measurements at end-expiration. Continuous assessment of CO was measured using the modified thermodilution technique provided by the PAC manufacturer and described elsewhere [25,26].

A thermister-tipped arterial PiCCO catheter (Pulsiocath 5F, 20 cm, PV2015L20; Pulsion Medical Systems AG, Munich, Germany) was placed in the descending aorta and connected to a bedside PiCCO plus monitor. Cardiac index and volumetric variables were measured with the single-indicator transpulmonary thermodilution technique. The PiCCO values were obtained by repeated injections of 15- or 20-mL boluses of ice-cold normal saline via a central line. The mean value of three consecutive measurements was used for analysis. If the difference between the three obtained values for cardiac index was greater than 10%, two additional measurements were performed subsequently. Finally, the mean of all consecutive measurements was used.

By means of the thermodilution curve, the PiCCO calculates CO by the modified Stewart-Hamilton equation, the mean transit time (MTt), and the exponential downslope time (DSt) of the curve. The product of CO times MTt gives the intrathoracic thermal volume (ITTV) [12,27]. The product of CO times the DSt gives the pulmonary thermal volume (PTV) [12,28,29]. The difference between ITTV and PTV is called global end-diastolic volume (GEDV), or GEDVI if indexed for the body surface area.

Stroke volume (SV) is calculated by dividing CO by heart rate. A 'global' ejection fraction (GEF) can be obtained by dividing SV by a quarter of GEDV. Similarly, dividing CO by the preload parameter GEDV gives an indicator of cardiac systolic function, the so-called CFI. Both GEF and CFI may provide information on left ventricular systolic function [30]. In patients with shock and multi-organ failure, GEF and CFI correlated closely with left ventricular fractional area of change using echocardiography [30].

The PiCCO method and definitions of intrathoracic blood volume and EVLW are described in more detail elsewhere [31]. For this study, EVLW was indexed to predicted body weight (ELWI), as proposed by Phillips and colleagues [16].

The ratio of EVLW to pulmonary blood volume is used as an index of pulmonary vascular permeability (PVPI). Additionally, we calculated the ratio of EVLW indexed for body weight to GEDVI (that is, ELWI/GEDVI × 10^2^) as another index of pulmonary vascular permeability [13].

In addition to mean arterial pressure (MAP), heart rate, continuous CO, and right atrial pressure, the following hemodynamic parameters were simultaneously recorded four times in each patient: cardiac index by both methods, mixed venous oxygen saturation (SmvO_2_), left ventricular stroke work index (LVSWI), PAOP, GEDVI, CFI, GEF, ELWI, and PVPI. For each recording, all variables were determined within 10 minutes. LVSWI was calculated by the formula SVI × (MAP – PAOP) × 0.0136, where SVI denotes SV index (SV divided by body surface area). For comparison purposes, we also calculated cardiac power (CP) using the formula CP = MAP × CO/451. The CP has been described as a valuable marker of outcome in patients with cardiogenic shock [32-34]. Definitions are provided in Table 2.

**Table 2:** Definitions

LVSWI = SVI × (MAP – PAOP) × 0.0136
CP = MAP × CO/451
ITTV = CO × MTt
PTV = CO × DSt
GEDV = ITTV - PTV = CO × (MTt - DSt)
ITBV = 1.25 × GEDV
CFI = (CO/GEDV) × 10^3^
GEF = SV/(GEDV/4)
EVLW = ITTV - ITBV
PVPI = EVLW/PBV

The detailed pathophysiological background is explained in Materials and methods. CFI: cardiac function index; CO: cardiac output; CP: cardiac power; DSt: exponential downslope time; EVLW: extravascular lung water; GEDV: global end-diastolic volume; GEF: global ejection fraction; ITBV: intrathoracic blood volume; ITTV: intrathoracic thermal volume; LVSWI: left ventricular stroke work index; MAP: mean arterial pressure; MTt: mean transit time; PAOP: pulmonary artery occlusion pressure; PBV: pulmonary blood volume; PTV: pulmonary thermal volume; PVPI: pulmonary vascular permeability index; SV: stroke volume; SVI: stroke volume indexed for body surface area.

### Statistical analysis

Clinical data were collected from the patients' charts, anonymised, and entered into a computerised database. Medians, 25th–75th percentiles (IQR), or percentages were calculated for the overall sample and subgroups. Comparisons between groups were made with the use of the Mann-Whitney *U *test or the Fisher exact test, as appropriate. Repeated measures within groups were compared with a Wilcoxon signed rank sum test. Including all consecutive hemodynamic measurements per patient, correlations between data pairs were established using linear regression analysis and are expressed as the square of Pearson's correlation coefficients (*r*^2^). To investigate the relationship between the cardiac index measured by PAC and PiCCO, bias and limits of agreement of data pairs were calculated as described by Bland and Altman [35]. Bias represents the systemic error between the two methods. Upper and lower limits of agreement, calculated as mean bias ± two standard deviations, define the range in which 95% of the differences are expected. The percentage error was calculated as 100 × (CO indexed for body surface area [CI] by PiCCO - CI by PAC)/[(CI by PiCCO + CI by PAC)/2], as proposed by Rödig and colleagues [36]. All analyses were performed using SPSS version 12.0 for Windows (SPSS Inc., Chicago, IL, USA). Statistical significance was defined as *P *values of below 0.05, and all hypothesis testing was two-tailed.

## Results

The clinical characteristics of the two study groups are described above. Net fluid balance during the 24-hour observation period was +2,066 (375 to 2,749) mL in septic patients as compared with +60 (-596 to 1,622) mL in patients with AHF (*P *= 0.11). ICU lengths of stay were 17 (14 to 30) days in septic patients and 12 (5 to 19) days in AHF patients (*P *= 0.13). Overall ICU mortality rates were 44% (4/9) among patients with sepsis and 25% (3/12) among those with AHF (*P *= 0.40).

### Hemodynamic measurements

Measurement of PAOP was unavailable in two AHF patients, and the SmvO_2 _was missing in another AHF patient. Hemodynamic measurements obtained at the first and fourth recordings are shown in Table 3. Between the first and the forth measurements, hemodynamic variables remained nearly unchanged. Exceptions were LVSWI (increase in septic patients), PAOP and GEDVI (decrease in AHF patients), and SmvO_2 _(increase in AHF patients). According to the mild changes during the observation period, we pooled the results within each group for further correlation purposes (Table 4). In comparison with patients with AHF, those with sepsis had higher cardiac index, CP, LVSWI, SmvO_2_, CFI, GEF, PVPI, and ELWI/GEDVI ratio but a lower PAOP. ELWI was higher in patients with sepsis, but this trend did not reach statistical significance (*P *= 0.09).

**Table 3:** Comparing the first and the fourth hemodynamic measurements in patients with sepsis and acute heart failure

	Sepsis		Acute heart failure	
	
	1st measurement	4th measurement	1st measurement	4th measurement
	n = 9	n = 9	n = 12	n = 12
Basic monitoring				
Heart rate, 1/minute	83 (80–96)	90 (78–108)	87 (72–97)	85 (75–95)
MAP, mm Hg	75 (65–81)	77 (69–86)	74 (70–78)	66 (58–75)^a, b^
RAP, mm Hg	13 (9.0–17)	12 (10–15)	13 (9.5–15)	11 (8.3–15)
SaO_2_, percentage	94 (91–96)	94 (93–97)	95 (94–96)	94 (93–97)
				
PAC				
CI, L/minute per m^2^	3.8 (3.1–4.3)	4.8 (3.7–6.2)	2.6 (1.9–3.1)^a^	2.8 (2.3–3.1)^a^
CP, W	1.00 (0.88–1.32)	1.33 (1.04–1.87)	0.71 (0.59–0.95)	0.80 (0.65–0.93)^a^
LVSWI, g-m/m^2^	32 (23–45)	42 (34–54)^b^	20 (17–25)^a, c^	18 (17–29)^a, c^
MPAP, mm Hg	31 (30–32)	27 (27–31)	32 (27–37)	31 (22–35)
PAOP, mm Hg	18 (15–21)	17 (14–18)	21 (17–26)^c^	19 (12–20)^b, c^
SmvO_2_, percentage	68 (61–74)	70 (60–75)	50 (48–62)^a, d^	59 (52–64)^a, b, d^
				
PiCCO				
CI, L/minute per m^2^	3.6 (3.5–5.6)	4.8 (3.8–5.4)	2.9 (1.8–3.8)^a^	2.9 (2.2–3.3)^a^
CFI, 1/minute	6.0 (3.3–6.8)	6.4 (3.5–8.0)	2.7 (2.2–2.9)^a^	2.7 (2.4–3.6)^a^
GEF, percentage	21 (15–30)	25 (16–34)	13 (9.8–14)^a^	14 (12–17)^a^
GEDVI, mL/m^2^	857 (703–1,128)	797 (660–1,164)	1,141 (893–1,311)^a^	904 (796–1,144)^b^
ELWI, mL/kg	18.2 (14.7–24.0)	15.3 (12.4–20.2)	15.4 (13.7–22.4)	15.7 (14.0–19.2)
PVPI	2.6 (2.2–4.1)	3.0 (1.9–3.4)	2.8 (2.0–3.8)	2.5 (1.9–2.8)
ELWI/GEDVI, × 10^2^	2.0 (1.6–2.7)	2.1 (1.4–2.3)	1.6 (1.2–2.4)	1.9 (1.4–2.2)

The results are presented as medians (interquartile ranges). ^a^*P *< 0.05 between the two groups for a given time point; ^b^*P *< 0.05 for changes between the two time points within a group. Reduced numbers because of missing data are indicated by superscript c (^c^), where n = 10, and superscript d (^d^), where n = 11. CFI: cardiac function index; CI: cardiac index; CP: cardiac power; ELWI: extravascular lung water index; GEDVI: global end-diastolic volume index; GEF: global ejection fraction; LVSWI: left ventricular stroke work index; MAP: mean arterial pressure; MPAP: mean pulmonary arterial pressure; PAC: pulmonary artery catheter; PAOP: pulmonary artery occlusion pressure; PiCCO: transpulmonary thermodilution technique; PVPI: pulmonary vascular permeability index; RAP: right atrial pressure; SaO_2_: arterial oxygen saturation; SmvO_2_: mixed venous oxygen saturation.

**Table 4:** Comparing hemodynamic characteristics between patients with sepsis and acute heart failure

	Sepsis n = 36	Acute heart failure n = 48	*P *value
PAC			
CI, L/minute per m^2^	4.2 (3.6–5.5)	2.6 (2.2–3.0)	< 0.001
CP, W	1.14 (0.99–1.63)	0.80 (0.62–0.94)	< 0.001
LVSWI, g-m/m^2^	38 (30–49)	23 (18–29)^a^	< 0.001
RAP, mm Hg	13 (9–15)	12 (8–14)	0.26
PAOP, mm Hg	16 (15–18)	20 (15–24)^a^	0.008
MPAP, mm Hg	29 (26–32)	32 (26–37)	0.02
SmvO_2_, percentage	68 (62–74)	57 (50–62)^b^	< 0.001
PiCCO			
CI, L/minute per m^2^	4.6 (3.7–5.6)	2.7 (2.2–3.3)	< 0.001
CFI, 1/minute	6.1 (3.5–6.8)	2.8 (2.3–3.1)	< 0.001
GEF, percentage	23 (17–30)	14 (10–16)	< 0.001
GEDVI, mL/m^2^	907 (748–1133)	995 (849–1172)	0.16
ELWI, mL/kg	18.0 (14.3–23.1)	14.7 (13.1–18.5)	0.09
PVPI	2.8 (2.3–3.5)	2.4 (1.7–2.9)	0.01
ELWI/GEDVI, × 10^2^	2.0 (1.7–2.4)	1.6 (1.3–2.2)	0.01

Data represent the median (interquartile range) of the four consecutive measurements obtained during the observation period. Reduced numbers because of missing values are indicated with superscript a (^a^), where n = 40, and superscript b (^b^), where n = 44. CFI: cardiac function index; CI: cardiac index; CP: cardiac power; ELWI: extravascular lung water index; GEDVI: global end-diastolic volume index; GEF: global ejection fraction; LVSWI: left ventricular stroke work index; MPAP: mean pulmonary arterial pressure; PAC: pulmonary artery catheter; PAOP: pulmonary artery occlusion pressure; PiCCO: transpulmonary thermodilution technique; PVPI: pulmonary vascular permeability index; RAP: right atrial pressure; SmvO_2_: mixed venous oxygen saturation.

A Bland-Altman analysis of cardiac index measurements by PiCCO and PAC resulted in a mean bias of 0.19 L/minute per square metre. Limits of agreement were -0.97 and 1.35 L/minute per square metre. The median percentage error of comparisons was 2.5%. It was within 15% for 68% of comparisons between CI by PiCCO and CI by PAC. In septic patients, *r*^2 ^between the two cardiac indexes was 0.81 (*P *< 0.001) compared with 0.58 in patients with AHF (*P *< 0.001).

**Figure 1:**
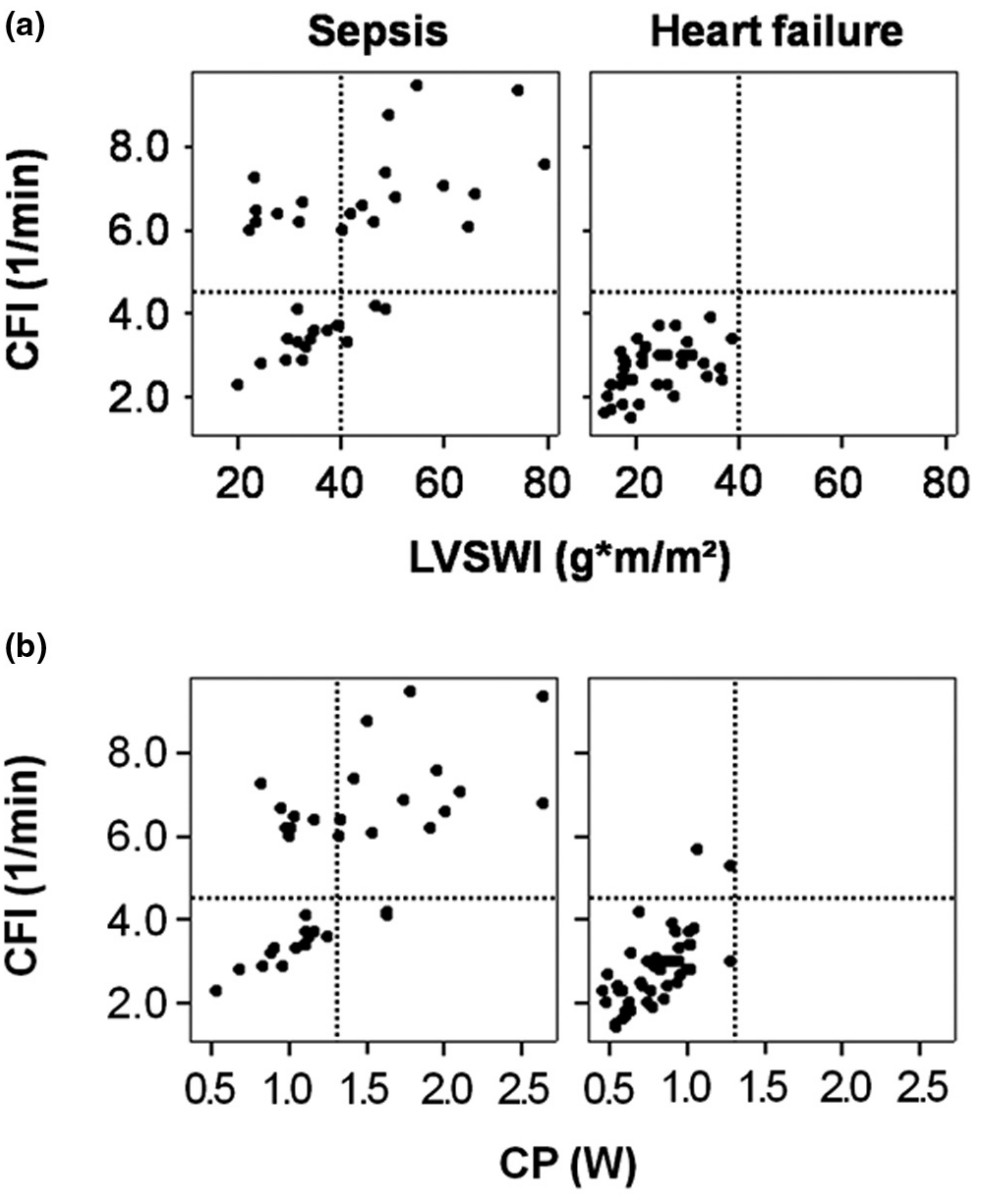
The relation between CFI, LVSWI and CP. **(a) **The relationship between cardiac function index (CFI) and left ventricular stroke work index (LVSWI) in patients with sepsis and those with acute heart failure. Significant correlations between the two variables exist in patients with sepsis (*r*^2 ^= 0.30, *P *= 0.001) and those with acute heart failure (*r*^2 ^= 0.23, *P *= 0.002). **(b) **The significant relationship between CFI and cardiac power (CP) in both patient groups (sepsis: *r*^2 ^= 0.39, *P *< 0.001; acute heart failure: *r*^2 ^= 0.45, *P *< 0.001). Dashed lines indicate CFI of 4.5 per minute, LVSWI of 40 g-m/m^2^, and CP of 1.3 W.

**Figure 2:**
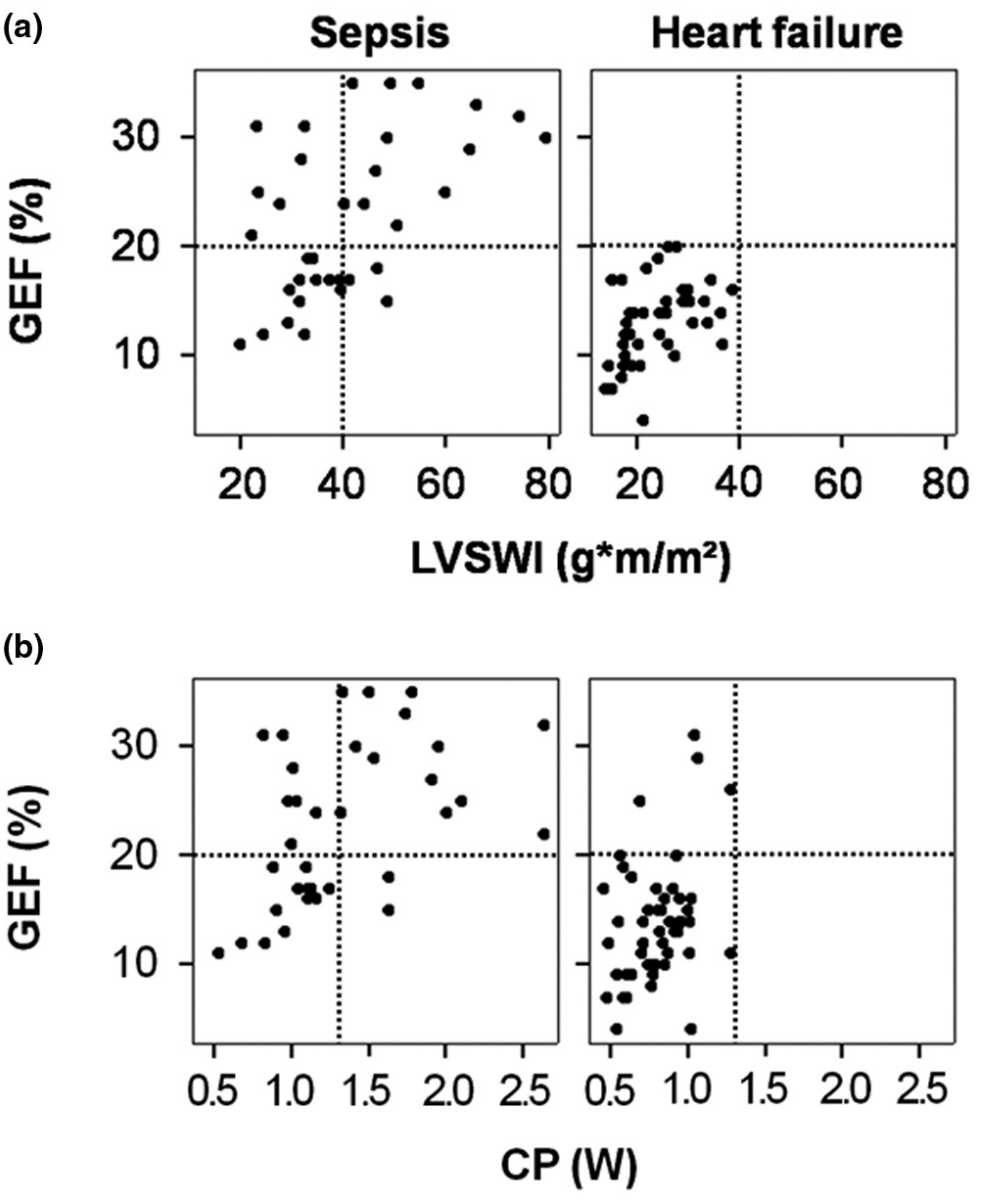
The relation between GEF, LVSWI and CP. **(a) **The relationship between global ejection fraction (GEF) and left ventricular stroke work index (LVSWI) in patients with sepsis and those with acute heart failure. Significant correlations between the two variables exist in patients with sepsis (*r*^2 ^= 0.26, *P *= 0.001) and those with acute heart failure (*r*^2 ^= 0.18, *P *= 0.006). **(b) **The significant relationship between GEF and cardiac power (CP) in both patient groups (sepsis: *r*^2 ^= 0.22, *P *= 0.004; acute heart failure: *r*^2 ^= 0.13, *P *= 0.01). Dashed lines indicate GEF of 20%, LVSWI of 40 g-m/m^2^, and CP of 1.3 W.

**Figure 3:**
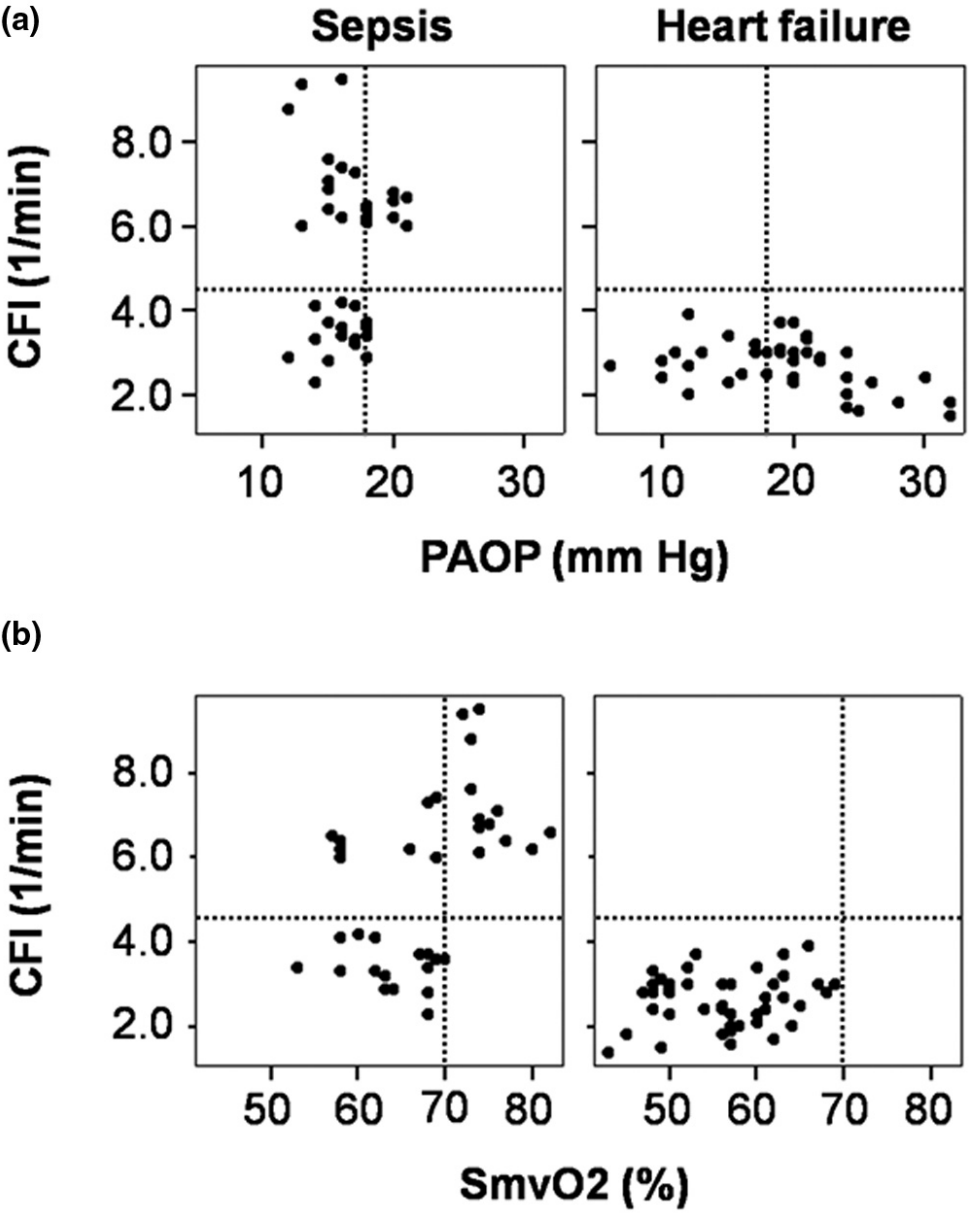
The relation between CFI, PAOP and SmvO2. **(a) **The relationship between cardiac function index (CFI) and pulmonary artery occlusion pressure (PAOP) in patients with sepsis and acute heart failure. In patients with acute heart failure, CFI is negatively correlated with PAOP (*r*^2 ^= -0.18, *P *= 0.006), whereas there is no significant correlation in septic patients (*r*^2 ^= 0.0006, *P *= 0.89). **(b) **CFI is significantly correlated with mixed venous oxygen saturation (SmvO_2_) in patients with sepsis (*r*^2 ^= 0.22, *P *= 0.004) but not in patients with acute heart failure (*r*^2 ^= 0.03, *P *= 0.26). Dashed lines indicate CFI of 4.5 per minute, PAOP of 18 mm Hg, and SmvO_2 _of 70%.

Comparisons between CFI and other markers of cardiac performance (CP and LVSWI) are displayed in Figure 1. Figure 1 demonstrates the significant correlation between CFI and LVSWI (sepsis: *r*^2 ^= 0.30, *P *= 0.001; AHF: *r*^2 ^= 0.23, p = 0.002) as well as CFI and CP (sepsis: *r*^2 ^= 0.39, *P *< 0.001; AHF: *r*^2 ^= 0.45, *P *< 0.001) in both patient groups. In Figures 1a and 1b, the CFI values of four septic patients with depressed cardiac function can easily be identified. The correlations between GEF and LVSWI (sepsis: *r*^2 ^= 0.26, *P *= 0.001; AHF: *r*^2 ^= 0.18, *P *= 0.006) plus GEF and CP (sepsis: *r*^2 ^= 0.22, *P *= 0.004; AHF: *r*^2 ^= 0.13, *P *= 0.01), as shown in Figure 2, were comparable to the corresponding CFI correlations in Figure 1. The overall correlation between CFI and GEF was *r*^2 ^= 0.81 (*P *< 0.001). Figure 3 shows the relationships between CFI and PAOP and between CFI and SmvO_2_. It demonstrates the significant negative correlation between CFI and PAOP (*r*^2 ^= -0.18, *P *= 0.006) in AHF patients but not in patients with sepsis (*P *= 0.89). On the other hand, CFI was significantly correlated with SmvO_2 _in septic patients (*r*^2 ^= 0.22, *P *= 0.004) but not in those with heart failure (*P *= 0.26).

All AHF patients had an SmvO_2 _of not more than 70% and a CFI of less than 4.5 per minute, except one suffering from congenital heart disease, who presented with low central venous oxygen saturation and shock. In this patient (classified as AHF because of her history), PAC showed a CI of 3.6 L/minute per square metre and an SmvO_2 _of 68%. PiCCO revealed a CFI of at least 4.5 per minute in two of four measurements and a GEF of greater than 20% in all four measurements.

All septic patients with cardiac dysfunction (CFI of less than 4.5 per minute) had an SmvO_2 _of not more than 70%. Among those with a CFI of at least 4.5 per minute, 1 patient had four SmvO_2 _values of less than 70%, most likely because of hypovolemia. The remaining four points of less than 70% (2 patients) were associated with an arterial oxygen saturation of below 92% (Figure 3b).

**Figure 4:**
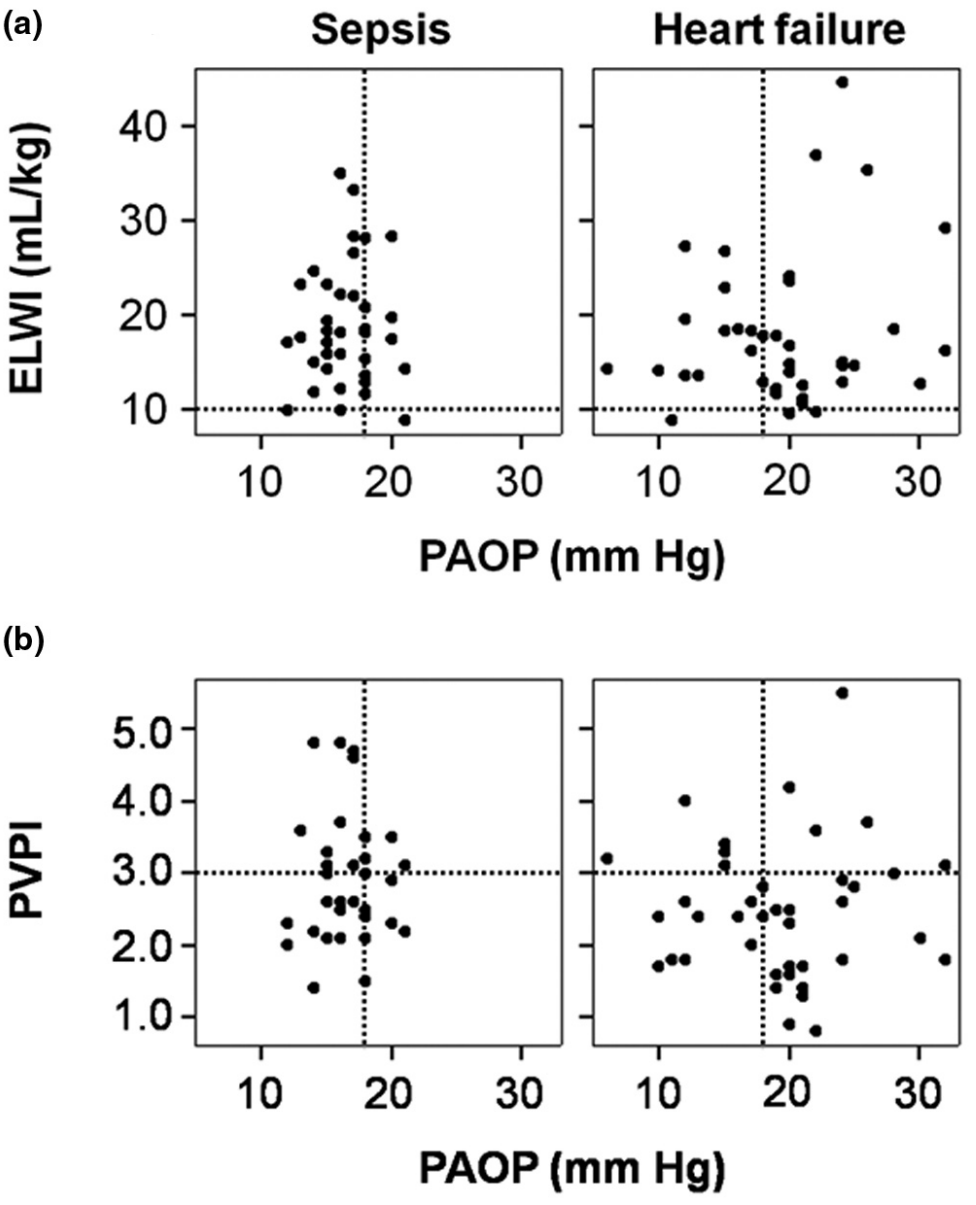
**The relation between PAOP, ELWI and PVPI**. **(a) **The relationship between pulmonary artery occlusion pressure (PAOP) and extravascular lung water index (ELWI) in patients with sepsis and patients with acute heart failure. **(b) **The relationship between PAOP and pulmonary vascular permeability index (PVPI) in patients with sepsis and patients with acute heart failure. No significant correlation exists between PAOP and the other two variables in either group of patients. Dashed lines indicate PAOP of 18 mm Hg, ELWI of 10 mL/kg, and PVPI of 3.0.

PAOP did not correlate with ELWI and PVPI either in septic or in heart failure patients (Figure 4). Five of 12 patients with AHF and 6 of 9 with sepsis had at least one PVPI value of greater than 3.0, indicating that PVPI may not discriminate between heart failure and sepsis. No correlations were found between PAOP and GEDVI (data not shown).

## Discussion

The results of the present study indicate that in patients with AHF and severe sepsis or septic shock the PiCCO-derived cardiac function parameters, namely CFI and GEF, are valuable and comparable to the more classic PAC-derived parameters such as CP and LVSWI and are better than PAOP and SmvO_2_. In patients with sepsis, the PVPI and the ratio of ELWI to GEDVI were only slightly higher than in those with AHF, which suggests an increased pulmonary vascular permeability in the latter group. Elevated ELWI despite a relatively low PAOP for patients with AHF supports this assumption.

Our results confirm that PiCCO-derived CO measurements parallel values obtained by PAC [20,37,38]. In addition to previous reports from surgical and septic patients, our data prove that this is also the case for critically ill medical patients presenting with AHF and a low CO. The systemic error (bias) of CO measurements between PiCCO and PAC was considerably lower in our medical ICU population. As previously reported, CO is usually slightly overestimated when measured in the aorta compared with the pulmonary artery [20,39].

SmvO_2 _is considered a surrogate marker of CO in several conditions [40]. In our study, we found that SmvO_2 _correlated with CFI in patients with sepsis but not in those with AHF. All patients presenting with AHF had an SmvO_2 _of below 70%. Among the septic patients with a CFI of greater than 4.5 per minute, three had SmvO_2 _measurements of below 70%. This observation is in line with the hypothesis that SmvO_2 _has a low sensitivity and specificity for the detection of myocardial dysfunction in patients with distributive shock [41-43]. Our results favour CO measurements over SmvO_2 _assessments for the monitoring of cardiac performance in septic patients.

PAC-derived LVSWI and particularly CP, the product of CO and MAP, are predictors of outcome in cardiogenic shock patients [32-34]. In our study, we found a good correlation between CFI and both LVSWI and CP, independently of whether patients had sepsis or AHF. Of note, the median left ventricular ejection fractions were below 30% in heart failure patients and normal in septic patients. The PiCCO parameters CFI and GEF have previously been shown to be reliable markers of left ventricular function when compared with echocardiographic assessments [27] and left ventricular dP/dt max [44]. Interestingly, CFI and GEF identified a subpopulation of septic patients with a myocardial function as poor as in AHF patients. The CFI cutoff level for a depressed myocardial function in our septic population was between 4 and 5 per minute, which is in agreement with the results of a recent study indicating that a CFI of less than 4 per minute estimated a left ventricular fractional area of change of less than 40% with a sensitivity of 86% and a specificity of 88% [30].

In AHF patients but not in septic patients, PAOP was negatively correlated with CFI, suggesting that PAOP in heart failure is a marker of myocardial dysfunction. This is in line with earlier results in patients with acute myocardial infarction [45], in which a PAOP of at least 18 mm Hg was associated with an increased mortality [46]. Caution is recommended when using PAOP as a surrogate marker of cardiac function because PAOP depends on left ventricular filling volume and compliance. Hence, the relationship between the left ventricular filling pressure and volume is not linear [47]. Therefore, other pressure-independent hemodynamic markers of cardiac function such as CFI, GEF, or CP are superior. The results of our study in septic and AHF patients suggest that CFI adequately reflects cardiac function and may be preferred to PAOP, LVSWI, and CP.

Consistent with previous studies in heart failure [8] and septic [48,49] patients, PAOP correlated neither with GEDVI nor with EVLW. In contrast to a recent study performed in patients with hydrostatic pulmonary edema and acute lung injury/acute respiratory distress syndrome (ALI/ARDS) [13], we could not find a significant difference in GEDVI and EVLW between heart failure and septic patients. However, we found a lower PVPI and ELWI/GEDVI ratio in patients with AHF than in those with sepsis. This is in accordance with the hydrostatic origin of pulmonary edema in the former group. In our septic patients, GEDVI was higher and ELWI and PVPI were both lower than in the patients with ALI/ARDS reported by Monnet and colleagues [13]. This difference may be explained by lower pulmonary vascular permeability and milder pulmonary edema in our patients. The PaO_2_/FiO_2 _(partial arterial oxygen pressure/inspired oxygen fraction) ratios were an average of 165 mm Hg in our septic patients and 118 mm Hg in patients with ALI/ARDS reported by Monnet and colleagues. In our AHF patients, PVPI and the ELWI/GEDVI ratio were surprisingly high, suggesting an increased pulmonary vascular permeability in addition to an elevated left ventricular filling pressure. These results add further evidence against the use of PAOP as the only criterion to differentiate between a hydrostatic and a permeability type of pulmonary edema [12,50,51].

The number of patients limits the results of our study. However, consistently using four measurements per patient in a condition close to a steady state over a short observation period of an average of 19 hours, 2 days after ICU admission, at least partially compensated for this limitation. Moreover, consecutive measurements in the same patients may have multiplied the number of errors. However, as seen in Tables 3 and 4, the IQRs for a single variable between the four sets of measurements and within the groups were small. Thus, our measurements made in two different and well-characterised clinical conditions probably give a realistic hemodynamic picture of the two populations, allowing a fair comparison between PiCCO and PAC. Another important point is that some of the investigated parameters are mathematically coupled. For example, LVSWI, CFI, and CP are all linked to SV. Similarly, GEDV is the preload index for both CFI and GEF. This fact might explain at least some of the significant correlations found in this study.

## Conclusions

The results of our study indicate that hemodynamic variables derived from the transpulmonary thermodilution method allow hemodynamic characterisation of patients with AHF and sepsis. In particular, a low CFI and GEF identified cardiac dysfunction in patients with AHF and in patients with severe sepsis or septic shock. Prospective studies are now needed to demonstrate that the PiCCO technology is superior to a standard of care based on the current recommendations for hemodynamic monitoring and management in shock [40,52].

## Key messages

• Pulse contour cardiac output (PiCCO)-derived cardiac output measurements parallel values obtained by pulmonary artery catheter, even in critically ill medical patients presenting with a low cardiac output.

• The PiCCO-derived cardiac function index and global ejection fraction are valuable parameters of cardiac function in patients with acute heart failure and severe sepsis or septic shock.

## Abbreviations

AHF: acute heart failure; ALI/ARDS: acute lung injury/acute respiratory distress syndrome; CFI: cardiac function index; CI: cardiac output indexed for body surface area; CO: cardiac output; CP: cardiac power; DSt: exponential downslope time; ELWI: extravascular lung water indexed for predicted body weight; EVLW: extravascular lung water; GEDV: global end-diastolic volume; GEDVI: global end-diastolic volume indexed for body surface area; GEF: global ejection fraction; ICU: intensive care unit; IQR: interquartile range; ITTV: intrathoracic thermal volume; LVSWI: left ventricular stroke work index; MAP: mean arterial pressure; MTt: mean transit time; PAC: pulmonary artery catheter; PAOP: pulmonary artery occlusion pressure; PiCCO: pulse contour cardiac output; PTV: pulmonary thermal volume; PVPI: pulmonary vascular permeability index; SmvO_2_: mixed venous oxygen saturation; SV: stroke volume; SVI: stroke volume indexed for body surface area.

## Competing interests

MM is a member of the Pulsion Medical Systems AG Medical Advisory Board. He received reimbursements for travel costs by the company for attending advisory board meetings and giving talks on several occasions. He received no grants for this study. The other authors declare that they have no competing interests.

## Authors' contributions

SR was responsible for data collection, carried out the statistical analysis, contributed to the interpretation of data, and drafted and revised the manuscript. AR carried out the statistical analysis, contributed to the interpretation of data, and revised the manuscript. MM developed the study design, coordinated data collection, helped to carry out the statistical analysis and interpretation of data, and revised the manuscript. All authors read and approved the final manuscript.
